# Predator Bounties in Western Canada Cause Animal Suffering and Compromise Wildlife Conservation Efforts

**DOI:** 10.3390/ani5040397

**Published:** 2015-10-19

**Authors:** Gilbert Proulx, Dwight Rodtka

**Affiliations:** 1 Alpha Wildlife Research & Management Ltd., 229 Lilac Terrace, Sherwood Park, AB T8H 1W3, Canada; 2 Alberta Agriculture, Problem Wildlife Specialist—retired, Rocky Mountain House, AB T4T 2A2, Canada; E-Mail: dgrodtka@gmail.com

**Keywords:** animal welfare, bounty, predators, shooting, snares, strychnine

## Abstract

Although predation bounty programs (rewards offered for capturing or killing an animal) ended more than 40 years ago in Canada, they were reintroduced in Alberta in 2007 by hunting, trapping, and farming organizations, municipalities and counties, and in 2009 in Saskatchewan, by municipal and provincial governments and the Saskatchewan Cattlemen’s Association. Bounty hunters use inhumane and non-selective killing methods such as shooting animals in non-vital regions, and killing neck snares and strychnine poisoning, which cause suffering and delayed deaths. They are unselective, and kill many non-target species, some of them at risk. Predator bounty programs have been found to be ineffective by wildlife professionals, and they use killing methods that cause needless suffering and jeopardize wildlife conservation programs. Our analysis therefore indicates that government agencies should not permit the implementation of bounty programs. Accordingly, they must develop conservation programs that will minimize wildlife-human conflicts, prevent the unnecessary and inhumane killing of animals, and ensure the persistence of all wildlife species.

## 1. Introduction

Bounties (rewards offered for capturing or killing animals) were commonly used throughout Europe from the 17th to 20th Century to control terrestrial predators, e.g., wolves (*Canis lupus*) [1], red foxes (*Vulpes vulpes*) [2,3], brown bears (*Ursus arctos*) [4,5], lynx (*Lynx lynx*) [6], otters (*Lutra lutra*) [7], pine martens (*Martes martes*) [6], and others. Not surprisingly, bounties were implemented in North America since European settlement. Virtually every American state or territory offered bounties at various times from the 1700s to the 1900s [8,9]. Five states still had wolf bounties on the legislative records as late as 1971 [10].

In Canada, wolf bounties occurred in the early 1700s [11], although the first documented bounty was in Upper Canada (Ontario) in 1793 [12,13]. By 1900, all Canadian provinces with wolves had bounties [14]. In the west, bounties started in 1878 in Manitoba, and 1899 in Saskatchewan and Alberta [15,16], and in 1900 in British Columbia. In Quebec and Ontario, bounties stopped in the early 1970s. Closure of bounties occurred in Saskatchewan in 1949, likely because of their ineffectiveness in controlling livestock depredation [17]. In most of Western Canada, however, bounty programs ended after the Predator Control Conference in Calgary in 1954 [16] e.g., that same year in Alberta and British Columbia, in 1965 in Manitoba, and in 1971 in Yukon.

As in the USA, most bounties by Western Canadian governments and stock grower associations were implemented to protect livestock. However, in the Canadian Territories, bounties were used for game management [14]. For some people, bounties were an excuse to get rid of “vermin” [18]. Predators have long been perceived as an important risk to humans for much of our history [19]. As a result, negative perceptions of the wolf make it difficult to find a compromise between human interests and wolf conservation [20]. Since the decimation by settlers and market hunters of native populations during the late 1800s, wolves and other large predators have killed livestock to survive, and the human determination to kill these carnivores has increased, and is still present today [20].

After centuries of bounty implementation, however, wildlife biologists realized that they were an ineffective management tool [21,22] that did not properly target or reduce predators [14], which maintained high population densities through compensatory reproduction and immigration into controlled areas. These bounty programs were also permeated with fraud [14]. Sometimes, adult female wolves in traps were released to maintain future reproduction, whereas trapped juveniles were occasionally held captive only to be killed later for adult bounty [8]. Counterfeit ears, or those of dogs (*Canis lupus familiaris*) and coyotes (*Canis latrans*), were submitted as proof of wolves killed [8,23,24]. Claims were also made for wolves taken outside the paying jurisdictions [8,25,26].

Most Canadian wildlife agencies no longer implement, support, or tolerate predator bounties. However, bounties for carnivores were reintroduced in Alberta in 2007, and in Saskatchewan in 2009, to protect livestock and increase the production of ungulates for hunters. Because people become bounty hunters to make as much money as possible, they use techniques that expedite the capture of target animals. However, we believe that their techniques have a significant impact on the welfare of target and non-target animals, and jeopardize wildlife conservation programs. In this paper, we show that bounties (1) are widely used in Western Canada; (2) are carried out with methods that cause animal suffering; and (3) are non-selective and may jeopardize the conservation of many species.

## 2. Methodology

The distribution of bounty programs in Alberta was determined through interviews with municipality and county officials, representatives of the provincial government, hunter and trapper associations, and with articles published in local newspapers. We collected data on bounty programs and the number of animals killed from 2010 to 2015. However, these are likely minimum estimates because of the apparent lack of rigor in data collection that we observed from one jurisdiction to the other. In Saskatchewan, only newspaper articles were used to determine the distribution of bounties from 2009 to 2015, and the number of animals killed during the 2009 to 2010 coyote bounty program.

The determination of methods used by bounty hunters to kill animals was based on discussions with government employees and members of hunter and trapper associations, information gathered from hunter and trapper interactive websites, scientific literature, and our own experience when visiting areas where bounties are being carried out. The review of the humaneness and selectivity of control methods used in bounty programs was based on scientific literature and our own field experience. The humane killing of wild animals requires that they die quickly, if not instantly, and with minimal pain [27,28]. Humane trapping devices must render canids irreversibly unconscious within 5 min according to the Agreement on International Humane Trapping Standards [29], but preferably within 3 min according to stricter standards [30].

## 3. Results and Review

### 3.1. Distribution of Bounties in Alberta and Saskatchewan

Bounties in Western Canada are being paid mostly for the control of wolves, coyotes, and cougars (*Felis concolor*). Jurisdictions with bounty programs represent approximately 20% and 50% of the surface areas of Alberta (total area: 661,848 km^2^) and Saskatchewan (651,900 km^2^), respectively.

#### 3.1.1. Alberta

In Alberta, any person or organization can, for any reason or no reason at all, offer a bounty on any species that can legally be killed. In rural counties, bounties may be offered to locals to incite them to kill rodents and predators that inhabit crops and grasslands. For example, American badgers (*Taxidea taxus*) may be hunted without a license, and during all seasons by a resident on privately owned land to which the resident has the right of access. Also, hunters are sometimes paid by local farmers to shoot American badgers [31].

**Figure 1:**
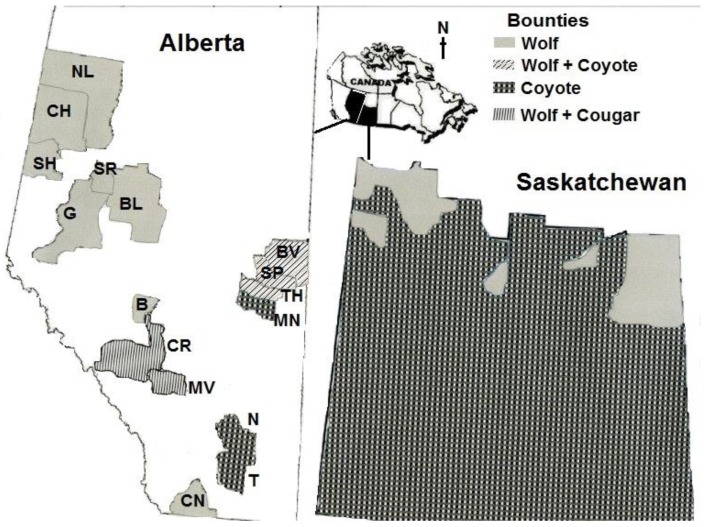
Areas with bounty programs in Alberta (2010–2015) and Saskatchewan (southern regions; 2009–2015). Alberta municipalities with bounties: Big Lakes (BL), Bonnyville (BV), Brazeau (B), Cardston (CN), Clearwater (CR), Clear Hills (CH), Greenview (G), Minburn (MN), Mountain View (MV), Newell (N), Northern Lights (NL), Saddle Hills (SH), Smoky River (SR), St. Paul (SP), Taber (T), Two Hills (TH).

In Alberta, wolf bounties are offered by municipal districts (rural municipalities that include either farmland, crown land, or a combination of both) [32] (Figure 1). Bounties range from $75 to $500 CAD, and at least 1425 wolves were reported killed by bounty hunters in the last five years (Table 1). In addition, Wild Sheep Foundation Alberta (WSF), a “wildlife conservation organization” promoting the interests of hunters, and Safari Club International (Red Deer Chapter; an organization promoting hunters’ rights and wildlife conservation), in conjunction with local trapper associations, offer a $300 CAD wolf bounty. They consider wolf predation on wildlife to be increasing at a high rate, and therefore causing a decline in ungulate populations; however, no scientific evidence has been provided to justify such a claim [32,33].

**Table 1:** Numbers of wolves and coyotes killed by bounty hunters since 2010 in Alberta municipalities and counties (see Figure 1 for geographic locations).

Municipality/County	Animals Killed by Bounty Hunters since 2010		Amounts Paid ($ CAN) per Dead Animal	
	Wolf	Coyote	Wolf	Coyote
Big Lakes (BL)	647	-	300	-
Bonnyville (BV)	30 since 2013	2200–2400 since 2013	75	15
Brazeau (B)	U ^**a**^	-	U	-
Cardston (CN)	16 since 2012	-	500	-
Clearwater (CR)	U		U	
Clear Hills (CH)	≥350		250–500	
Greenview (G)	90 since 2012	-	300	-
Minburn (MN)	U	240 since 2011	U	15
Mountain View	U		U	
Newell (N)	U	10,000 since 2010	U	15
Northern Lights (NL)	>185 since 2012	-	250	-
Saddle Hills (SH)	35 since 2011	-	500	-
Smoky River (SR)	12 since 2012		250	-
St. Paul (SP)	~60 since 2010	7500 since 2013	75	15
Taber (T)	U	2000–2500 since 2010	U	10
Two Hills (TH)	U	4000 since 2011	U	15

**^a^** Unknown.

In Alberta, bounties on coyotes (usually $15 CAD for each dead animal) are sponsored by municipal governments and local Fish and Game Associations [34,35] (Figure 1). At least 25,940 coyotes were reported killed by bounty hunters in 2010–2015 (Table 1). In Western Alberta, WSF offers a $4,000 CAD bounty on cougars killed in any Provincial Management Unit with bighorn sheep (*Ovis canadensis*) where the provincial cougar harvest quota has not been filled. This is to ensure that as many cougars as possible are killed annually. This initiative is part of their Ungulate Enhancement (Cougar Management 2013–2014) Project [36].

#### 3.1.2. Saskatchewan

In Saskatchewan, ranchers on the fringe of forested areas (Figure 1) can receive $250 CAD per dead wolf from rural municipal governments and the Saskatchewan Cattlemen’s Association [37,38,39].

In 2009–2010, Saskatchewan financed a province-wide coyote bounty program. Each dead coyote was worth $20 CAD. More than 71,000 coyotes were killed [40]. Because of this program, some coyotes were killed in the adjacent province of Alberta and submitted to the government of Saskatchewan for payment [41].

### 3.2. Animal Welfare Issues

Bounty hunters are known to use shooting, lethal trapping, and poisoning to kill as many animals as possible [42]. When trapping wolves, they prefer killing neck snares [43]. In Western Canada, liquid strychnine is available to farmers for the control of rodents, namely the northern pocket gopher (*Thomomys talpoides*) and the Richardson’s ground squirrel (*Urocitellus richardsonii*) [44]. Strychnine is easy to obtain, and some bounty hunters use it without special permits. Also, some Alberta Government biologists and university researchers use strychnine to poison wolves in culling experiments [45], thus suggesting that it is correct to use this poison to kill large predators although its use is biologically and ethically unacceptable [46,47].

#### 3.2.1. Shooting

Factors that can impact the humaneness of shooting include the skill of the shooter, the type of firearm, the type of ammunition, and most importantly, the point of impact of the bullet [48]. Shooting is considered humane when it causes direct destruction/concussion of brain tissue resulting in rapid unconsciousness, e.g., accurate shooting in the head [28]. However, as most hunters know, the best location for shooting free-ranging animals is the heart/lung region, rather than the head, because it presents a bigger target [30]. Unconcerned with the quality of the meat or the pelt, bounty hunters may shoot animals in non-vital regions such as the abdomen or the rump (authors’ personal observations). When animals are shot at, some will be killed outright, others will be missed, and some will be wounded but not killed. Of the ones that are wounded, some will be killed by subsequent shots but some will escape to either die later or recover, often with obvious debilitation [49].

#### 3.2.2. Killing Neck Snares

Killing neck snares are the most popular trapping devices used by bounty hunters because they are cheap, lightweight, easy to set and camouflage (except power snares), and efficient at capturing a diverse range of predators, namely wolves, coyotes, red foxes, cougars, and others. Killing neck snares are inadequate for consistently and quickly rendering canids unconscious [43]. Because of collateral blood circulation, it is almost impossible to stop blood flow to and from the brain by tightening a snare around the neck. Also, it is difficult to collapse the trachea due to its rigid cartilaginous rings and adjacent musculature [43]. Furthermore, weather conditions impact the function of snares, and the animals’ stride and posture when entering the loop affect capture location on the body [50]. Also, in an attempt to escape, animals frequently chew the snare, and cut their mouths and break their teeth [51]. If they do not escape, they then suffer a slow death with the snare embedded in their neck. Animals may develop a water or jelly head when not killed quickly, *i.e.*, an extreme case of edema due to watery fluid collecting in the tissues of the cervical region [52]. If they escape with the snare still closed on their neck, they may suffer for many days or weeks and eventually die with the snare cable cutting into their skin and muscles [53,54].

#### 3.2.3. Strychnine

Strychnine is a highly toxic alkaloid that causes suffering in poisoned animals [46]. It causes unimpeded stimulation of motor neurons affecting all the striated muscles of the body to produce generalized rigidity and tetanic seizures [55]. Affected animals often assume a “sawhorse stance” due to spams of the neck and back muscles causing extension of the head and neck, while spasms of the leg muscles cause the legs to become rigid and widely spread. Death by strychnine is inhumane because affected animals remain conscious and appear to suffer pain and anxiety from the onset of clinical signs until death [46], which can take up to 24 hours or longer if the dose is low [56]. Importantly, the use of strychnine is in contravention with all professional animal welfare guidelines [47].

### 3.3. Non-Selectivity and Conservation Issues

Bounties are the source of major concerns among wildlife professionals because the killing methods are non-selective, and they can jeopardize the conservation of many species.

Shooting allows bounty hunters to at least select the species that they intend to kill. With killing neck snares and strychnine poisoning, however, many non-target species are being killed. Killing neck snares are highly indiscriminate, killing species at risk as well as prey that the bounties aim to increase by reducing the densities of large predators [43].

Likewise, strychnine baits are non-selective and kill predators and scavengers. In Western Canada, strychnine poisoning has historically been responsible for the extirpation and endangerment of many species [47]. In southern Saskatchewan, as in many other regions of the world [57], strychnine is responsible for the death of many non-target species including birds of prey and species at risk [58]. Also, it caused a major decline in American badger populations [59], and this species now has the status of “Species of Special Concern” [60]. Although liquid strychnine is sold for the control of ground squirrels, it is also illegally used to kill predators [61]. In the Canadian Prairies, this could compromise the persistence of endangered swift foxes (*Vulpes velox*) and other species at risk (58). In Western Alberta, strychnine-laced baits also have the potential to kill wolverines (*Gulo gulo*), provincially listed as “May Be At Risk” [62], and grizzly bears (*Ursus arctos*), a provincially “Threatened” species [63].

## 4. Discussion

In a letter to the Premier of Alberta [64], the IUCN Canid Specialist Group requested, in vain, that the Government of Alberta stop indiscriminate wolf bounty programs by private groups and municipalities. Predator bounty programs employ inhumane and indiscriminate killing methods that impact on many non-target species, particularly those at risk. These bounties have all the attributes that should lead to their denunciation and abolition by government officials. Yet Alberta and Saskatchewan Fish and Wildlife Divisions have, in our view, abdicated their responsibility to properly manage all wildlife, target and non-target species, affected by bounties. Fish and Wildlife Divisions are stewards of natural resources for all citizens [65,66], and they should not permit the implementation of bounty programs to serve the interests of a few groups. Government agencies obviously need to better assess the real impact and the causes of predation on livestock and big game. Accordingly, they must develop conservation programs that will minimize wildlife-human conflicts, prevent the unnecessary and inhumane killing of animals, and ensure the persistence of all wildlife species.

Allowing predator bounties is very likely a political decision aimed at appeasing special interest groups such as the ranchers who have lost livestock, even though they already receive compensation from the government for their losses [67], and hunting groups who believe wolves and cougars are reducing the number of ungulates available for them. Indeed, politicians use bounties because they are means for them to provide their constituents with supplementary income, and to present themselves as defenders of peoples’ rights [68].

Failure of bounties to noticeably reduce and control coyote [22] and wolf [14] populations, and to significantly increase game species [69], is the main reason why bounties are not widely used today. Bounties are ineffective and inhumane, and their use is unjustified. Killing for “conservation” often proves to be unjustified because although the costs to those individuals killed are certain, the benefits to populations and ecosystems are not [70]. Bounties are similar to the wolf culling program that the Government of Alberta established in 2005 to recover threatened boreal caribou (*Rangifer tarandus*) populations [45]. The culling program was conducted with aerial shooting, snaring, and strychnine poisoning. Like bounties, the culling program was largely unsuccessful [45]. Although it gave the impression that something was being done to increase caribou numbers, the culling program diverted the public’s attention from the real problem at hand, *i.e.*, habitat loss and deterioration due to oil and gas and forestry industries. The scientific community, environmental organizations, and the public must vigorously condemn the use of such programs in Alberta and Saskatchewan.

It is important to educate the public, and particularly farmers, ranchers, and hunters about bounties, inhumane and indiscriminate killing methods, and responsible wildlife conservation and practice. There is a false but persistent belief among people that killing neck snares are humane [71]. However, the scientific community has all of the hard evidence to demonstrate that these trapping devices are neither humane nor selective, and should be treated with the same repugnance environmental groups did for the leg-hold trap. Likewise, the cruelty and non-selective characteristics of strychnine have been repeatedly denounced by scientists [47]. It is inconceivable to the authors that wildlife professionals would allow the use of these killing methods in the 21st Century.

## 5. Recommendations

In an effort to dispose of killing snares, the International Humane Trapping Standards must recognize the cruelty and ineffectiveness of these devices, and in the absence of humane killing snare technology, governments should phase out all killing neck snares [43]. The use of strychnine to kill fossorial rodents should be replaced by the implementation of Integrated Pest Management programs where predation, along with cultural, mechanical, and specific chemical techniques, are used as control agents [44]. Eliminating strychnine in agricultural pest control programs would minimize illegal use of the poison.

Better husbandry may help reduce human-wildlife conflicts. Ranchers should avoid leaving dead animals on the range, which attract predators and scavengers. They should only send healthy animals onto the range, along with dogs for protection from predators. Where feasible, animals should be kept close to the estate and be protected with surrounding fences. Instead of funding the killing of large predators, hunters wishing to increase deer (*Odocoileus* spp.), moose (*Alces alces*) and bighorn sheep should invest in habitat enhancement and restoration that would provide these species with food and protective cover against harsh weather and predators.

## 6. Conclusions

Bounties are ineffective to control predator populations, and they employ inhumane and indiscriminate killing methods. They should be made illegal in all jurisdictions. Predator management and conservation programs should be based on real field data and developed with rigorous animal welfare standards.
